# A Fluidic Biosensor Based on a Phase-Sensitive Low-Coherence Spectral-Domain Interferometer

**DOI:** 10.3390/s18113757

**Published:** 2018-11-03

**Authors:** Cuixia Guo, Xiaojie Yang, Zhiyuan Shen, Jian-Ping Wu, Suyi Zhong, Yonghong He, Tian Guan, Fangyi Chen

**Affiliations:** 1 Department of Physics, Tsinghua University, Beijing 100084, China; guo_c_x@163.com; 2 Shenzhen Key Laboratory for Minimal Invasive Medical Technologies, Institute of Optical Imaging and Sensing, Graduate School at Shenzhen, Tsinghua University, Shenzhen 518055, China; sueyi166@163.com; 3 Department of Biomedical Engineering, Southern University of Science and Technology, Shenzhen 518055, China; yangxj3@sustc.edu.cn (X.Y.); wujp@sustc.edu.cn (J.-P.W.); 4 Orbbec Co., Ltd., 12/F, No. 63 Xuefu Road, Nanshan District, Shenzhen 518055, China; shenzhiyuan@orbbec.com; 5 Academy of Advanced Interdisciplinary Studies, Southern University of Science and Technology, Shenzhen 518055, China; 6 School of Medicine, Tsinghua University, Beijing 100084, China; 7 Graduate School at Shenzhen, Tsinghua University, Shenzhen 518055, China; guocx14@mails.tsinghua.edu.cn

**Keywords:** phase-sensitive, low-coherence spectral-domain interferometer, fluidic, immunoassays

## Abstract

A phase-sensitive fluidic biosensor based on a spectral-domain low-coherence interferometer is presented in this paper. With a fiber optic probe employing the common-path interferometric configuration, subnanometric changes in thickness of the molecular layers can be detected through phase analysis of the acquired interference signal from the sensor surface. Advantages of this biosensor include its picometer-scale thickness sensitivity, 13.9-ms time response, and tolerance to the fluctuation in concentration of the target solution. The capabilities of this biosensor in monitoring specific molecular binding and recognizing specific molecular was successfully demonstrated by using the reactions between the molecules of protein A and IgG. The calculated minimum detectable concentration of IgG is 0.11 µg/mL.

## 1. Introduction

Techniques capable of molecular recognition and detecting molecular interactions play important roles in multiple fields such as medical diagnosis, drug screening, and environmental monitoring. These techniques can be divided into two categories, which include the label-free methods and the label-based methods. Compared with those label-based methods (e.g., chemiluminescence [1,2] and fluorescence [3,4,5]), which typically provide only the concentration of the target molecules, label-free detection methods are able to provide more information regarding the dynamics of the molecular reaction (e.g., molecular binding) and, therefore, reveal more features such as the molecular affinity, binding kinetics, inter-molecule adhesion, and concentration of the involved molecules [6,7]. Moreover, those label-based detection methods may cause undesired structural alterations and disturbance in reaction characters of the target molecules [8]. Complicated and time-consuming labeling procedures, in addition, are also required by the label-based methods. Therefore, more and more attention has been paid to label-free detection methods nowadays. 

The most prevalent label-free molecular detection methods include surface plasmon resonance (SPR) [9,10,11], gravimetric sensors [12,13], and the ones sensitive to electrochemical signals [14,15,16] or optical absorbance [17,18]. SPR detects the changes in the resonant angle or wavelength caused by the alteration of refractive index at the sensor surface and subsequently provides high-sensitivity data in real time. Electrochemical detection as a technique reliant upon the electrical signal is susceptible to the fluctuations in ion concentration and/or pH of the solution. Applications of the optical-absorbance method are usually limited by its unsatisfactory sensitivity due to the small volume and short optical path length of the sample. 

As one type of the label-free immunoassays detection technique, optical-interferometry methods have advantages such as high sensitivity, real-time detection, and the ability in characterizing molecular affinity and kinetics. The Mach-Zehnder interferometer (MZI) [19], integrated Young’s interferometry [20], and the optical biological compact disc (BioCD) [21] are the most commonly employed ones at present. They measured the change in thickness of the molecular layers induced by the binding of the target molecules onto the probe surface. Spectral domain low coherence interferometry (SD-LCI) employs coherence gating mechanism to acquire path-length-resolved interference information, which enables the extraction of thickness information only from the probe surface with the exclusion of interfering signals from other surfaces. Other advantages of the SD-LCI-based biosensors include their microsecond time response and tolerance to the fluctuation in concentration of the target solution.

In this study, we have developed an SD-LCI-based fluidic biosensor for label-free and real-time immunoassays. This biosensor consists of a fiber optic probe employing a common-path configuration (which acts as an in situ detector) and a fluidic chip with a built-in reaction cavity (which acts as a holder of the target solution). When the thickness of the molecular layer at the probe surface is changed by the binding of the target molecules, the optical path length of the sample arm would be altered. As a result, the phase of the sample-arm light and, subsequently, the phase of the interference signal will be changed. Therefore, this thickness change and, thus, molecular binding can be detected by using our biosensor and resolving the phase dynamics of the interference signal acquired from the probe surface. Moreover, the common-path design of the interferometer employed in our biosensor precluded the common-mode phase noise and, therefore, permitted the measurement to be of picometer-scale sensitivity. The efficacy of this developed fluidic biosensor has been experimentally demonstrated by the successful detection of a specific molecular binding reaction. The specifications such as the sensitivity and specificity of the developed biosensor were also measured in our study.

## 2. Materials and Methods

### 2.1. Preparation of the Reagents

The entire experimental procedure includes functionalization of the probe surface and label-free molecular sensing. The functionalization requires a protein-free blocking reagent (Baiaolaibo, Beijing, China), a protein A (Bioss, Beijing, China) solution (250 µg/mL in phosphate-buffered saline, PBS, Sigma-Aldrich, St. Louis, MO, USA), and a freshly prepared dopamine-Tris solution (2 mg/mL). For preparing the dopamine-Tris solution, we dissolved 12 mg Tris-HCl (0.1 mmol) into 10 mL deionized water and subsequently adjusted the pH to 8.5 by using a 10% HCl solution (Kaixin, Hengyang, China), which was followed by dissolving 0.02 g dopamine (Aladdin, Shanghai, China) in this solution. The dopamine-Tris solution must be prepared freshly because it is easily oxidized and blackened in air. For molecular sensing, immunoglobulin G (IgG, rabbit, Bioss, Beijing, China) solution at a concentration of 50 μg/mL in PBS buffer was prepared and sequentially diluted to concentrations of 30, 20, and 10 μg/mL.

### 2.2. Design and Fabrication of the Fiber Optic Probe

**Figure 1:**
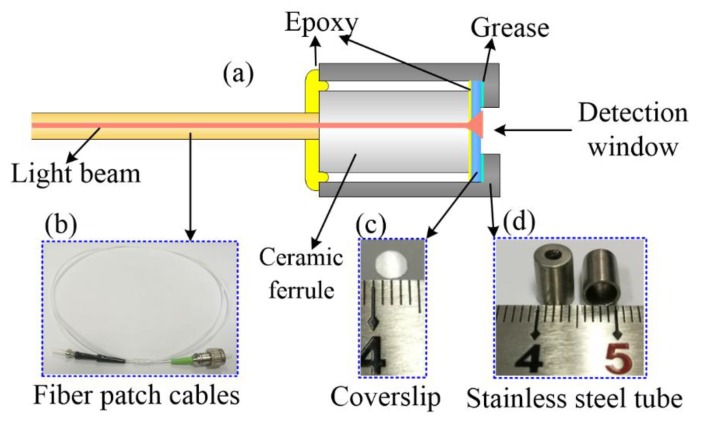
A schematic show of the fiber optic probe (**a**). The fiber optic probe consists of three main components: a fiber patch cable (**b**), a coverslip (**c**), and a stainless steel tube (**d**).

As shown in Figure 1, the fiber optic probe consisted of three main components including a single-mode-fiber patch cable, a coverslip, and a stainless-steel tube. All these components were carefully cleaned before the fabrication of the fiber optic probe. Specifically, the coverslip was immersed in piranha solution (sulfuric acid and hydrogen peroxide, volumetric ration 7:3) for three hours, which was followed by a rinse with deionized water and drying with nitrogen. The ceramic ferrule and the stainless-steel tube were both washed using ethanol (98%, Baishi, Tianjin, China), rinsed using deionized water, and then dried using nitrogen. In a fabricated fiber optic probe, the patch cable coupled the probe with the detection system via an FC/APC connector and, thus, delivered the incident light from the system to its probe end, which was embraced within a UPC-polished ceramic ferrule (diameter 2.5 mm, length 5 mm). A round coverslip (thickness 200 µm, diameter 3 mm) was further glued on the ceramic ferrule with epoxy. The ceramic ferrule and the embraced fiber probe as well as the coverslip were subsequently encased within a stainless-steel tube (length 7 mm, diameter 4 mm). A detection window (diameter 2 mm) located at the end of the tube permitted contact between the coverslip and the detected fluid. For firmly fixing the ceramic ferrule within the stainless-steel tube, epoxy was applied to seal the tail-side opening of the tube. Moreover, the space between the coverslip and the internal wall of the stainless-steel tube (e.g., induced by loose contact) was filled with a thin layer of grease to prevent possible influx of the detected fluid.

### 2.3. Design and Fabrication of the Fluidic Biosensor

**Figure 2:**
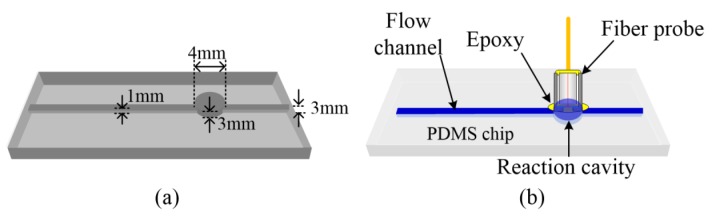
(**a**) A schematic show of the stainless-steel molding template (with positive structures) of the fluidic chip. (**b**) A schematic illustrating the fabricated fluidic biosensor with the fiber optic probe inserted into the reaction cavity.

The fluidic chip was molded with polydimethylsiloxane (PDMS) following a stainless-steel template. The molding template with positive structures is shown in Figure 2a. The PDMS base and curing agent (Sylgard 184; Dow Corning, Midland, MI, USA) were mixed (mass ratio 10:1), de-gassed, and then decanted on the template. After that, the mixture was thermally cured at 60 °C for three hours before the PDMS chip was peeled off from the template and bonded on a glass slide. For ensuring the permanent bonding between the PDMS and the glass slide, we treated the PDMS chip by exposing it to an oxygen plasma (Plasma Prep II, Structure Probe, West Chester, PA, USA) for 30 s before bonding with a glass slide. Such treatment guaranteed a robust fluidic chip that avoided fluid leakage even with the presence of high-velocity flow. The built-in structures, a flow channel (diameter 1 mm), and a reaction cavity (diameter 4 mm, depth 3 mm) were formed on the fluidic chip. The fiber optic probe, consequently, was inserted into the reaction cavity. Epoxy was again employed to seal the cylindrical interface between the probe and the cavity wall to prevent any possible air influx during the detection. A completed fluidic biosensor, consisting of a fluidic chip and a fiber optic probe, is shown in Figure 2b.

### 2.4. System Setup

**Figure 3:**
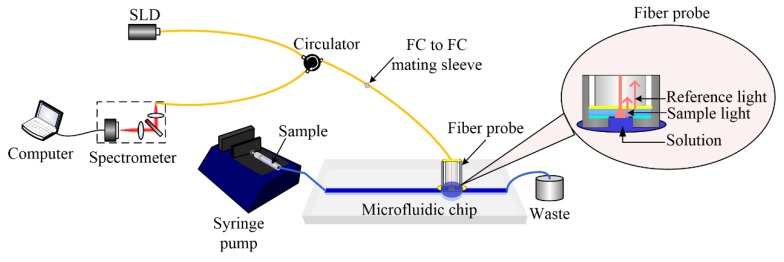
A schematic show of the detection system. SLD: superluminescent diode.

The customized detection system used in this study is shown in Figure 3. A super luminescent diode (SLD, IPSDS1307C, InPhenix, Inc., Livermore, CA, USA) acting as a light source provided the incident light with a central wavelength of 1310 nm and a bandwidth of 75 nm (at 3 dB). The incident light was delivered to the fiber optic probe via a circulator. The light interference required by the low coherence interferometry occurred between the back reflected light from the fiber-epoxy interface, which served as the reference light, and the back scattered light from the reflectors of coverslip-solution, which served as the sample light, when these two beams recombined within the circulator. Such a common-path design of the interferometer precluded the phase noise and, therefore, offered higher phase stability in comparison with the conventionally designed interferometer in which the reference arm and sample arm were separated. The equivalent lateral detection region is 9.2 µm (in air), which is approximately equal to the mode field diameter of the fiber because other light beams away from this area cannot reflect back to the single-mode fiber. The axial detection region is 17 µm in air, which is determined by the coherence length of the light source. The interference signal was sequentially collected by a homemade spectrometer where all the spectral components of the light source are spread out and detected simultaneously by a line-scan charge-coupled device (CCD) (GL2048R, Sensors Unlimited, Inc., Princeton, NJ, USA). The spectral resolution of this spectrometer was designed to be 0.06 nm. The line-scan rate of the CCD, which determines the sampling frequency of this biosensor, was 36 kHz. The real time analysis enabled by the customized data processing software written in C# allowed our detection system to specify the temporal dynamics of the signal phase, which carried valuable information for the following identification and quantification of the targeted molecules.

### 2.5. Data Processing and Analysis 

The obtained interference spectrum by the spectrometer can be expressed by the equation below.

(1) $$I\left(k\right)=S\left(k\right)\left(R_{r}+R_{s}+2\sqrt{R_{r}R_{s}}\cos\left(2k\left(z_{0}+nz\left(t\right)\right)\right)\right)\;$$

with the assumption that the spectrum of the light source follows a Gaussian spectral distribution, *S*(*k*). In Equation (1), *k* is the wavenumber and defined as $k=\frac{2\pi }{\lambda }$ where *λ* is the wavelength of the broadband source ranging from 1230 nm to 1360 nm. *R_r_* and *R_s_* are reflectivity values of the fiber-epoxy interface and the coverslip-solution reflector, respectively. *z*_0_ denotes the position of the sensor surface, *z*(*t*) denotes the thickness change induced by molecular absorption at the probe surface, and *n* denotes the refractive index of the molecular layer. After eliminating the DC part and resampling the spectrum, Fourier transform was applied to acquire the path-length-resolved interference signal, *I*(*z*), which can be calculated by using the equation below.

(2) $$I\left(z\right)=2\sqrt{R_{r}R_{s}}\Gamma \left(z-z_{0}\right)\exp\left(i\left(2k_{0}nz\left(t\right)\right)\right)\;$$

where Γ(z) is the inverse Fourier transform of the source spectrum *S*(*k*) and *k*_0_ is the source center wavenumber. 

The path-length-resolved intensity signal is used for locating a specific interference signal acquired from the molecules-coupled probe surface. The magnitude of such a signal is determined by and reveals the reflectivity values of the reference reflector and the sensor surface while the phase dynamics of such a signal can be calculated by using the equation below.

(3) $$\phi \left(t\right)=2k_{0}nz\left(t\right)\;$$

This reflects the change in thickness of the molecular layer at the probe surface. Molecular binding occurring at that surface, therefore, can be detected by monitoring the induced phase change at the probe surface. In our data processing, each phase value was averaged from 500 measurements to minimize the phase noise. Then the molecular thickness can be calculated by using the formula below.

(4) $$z(t)=\frac{\phi \left(t\right)}{2k_{0}n}=\frac{\phi \left(t\right)}{2.9k_{0}}\;$$

The assumption that the refractive index of the molecular layer is a constant of 1.45 was applied [22]. As the sampling frequency of the employed CCD camera was set to 36 kHz, the time response of the biosensor was 13.9 ms. The recovery time was equivalent to the time response in our developed system and, thus, also 13.9 ms. 

### 2.6. Experiments

Fluids were pumped through the flow channel using a syringe pump (Pump 11 Elite, Harvard Bioscience, Holliston, MA, USA). The flow rate of the solution injection was 200 μL/min. The temperature was controlled as 25.0 ± 0.3 °C during the entire experiment. All the involved reagents were stored in 25 °C water bath for 1 h, then immediately followed the detection. Functionalization of the sensor surface was performed before the detection of molecules binding. The functionalization includes three steps: first, depositing a thin dopamine adhesion layer onto the sensor surface in order to improve the biocompatibility of the probe [23], then, modifying the dopamine layer with the probe molecules of protein A, and, third, blocking the nonspecific binding sites on the dopamine layer using a protein-free blocking reagent. The small blocking molecules fill the gap between the protein A molecules and, thus, reduce nonspecific bindings of target molecules on the dopamine. PBS influx was introduced to the flow channel to flush the free molecules after each step. Activities of all these molecular modification and bindings were analyzed by specifying the phase dynamics of the interference signal. 

## 3. Results and Discussion

**Figure 4:**
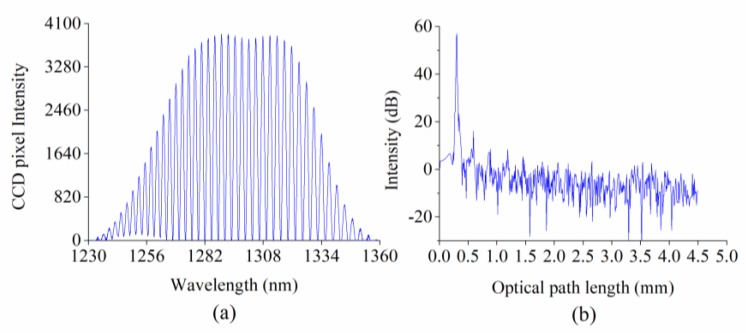
The background interference signal acquired with the presence of the PBS buffer solution presented at the (**a**) spectral domain and (**b**) the time/spatial domain.

Figure 4 shows the background interference signal obtained with the presence of PBS buffer at the spectral domain (Figure 4a) and time/spatial domain (Figure 4b), respectively. The interference signal at the spectral domain is an amplitude-modulated cosine signal generated by the light source following its spectral distribution function. The amplitude of the cosine signal corresponds to the signal peak at the time/spatial domain and manifests the SNR of the system. The exposure time of the CCD is adjusted in order to maximize the peak value of the cosine signal. As shown in Figure 4a, the valley value of the cosine signal was zero, which was achieved by adjusting the reference light intensity to approximately equal to the sample intensity. In this study, this adjustment was conducted by filling the air gap between the optical fiber and coverslip with epoxy, which is not only an adhesive but also a material that provides an appropriate reference reflectivity, *R_r_*, which satisfies $R_{r}=\frac{{\left(n_{1}-n_{2}\right)}^{2}}{{\left(n_{1}+n_{2}\right)}^{2}}$ (*n*_1_ and *n*_2_ are the refractive indices of the fiber and epoxy, respectively). When the peak value of the cosine signal is maximized and the valley value is approximately equal to zero, a maximum amplitude and, thus, a maximum SNR and minimum phase noise can be obtained. By this way, the performance of the biosensor was optimized with a measured SNR of 58 dB.

**Figure 5:**
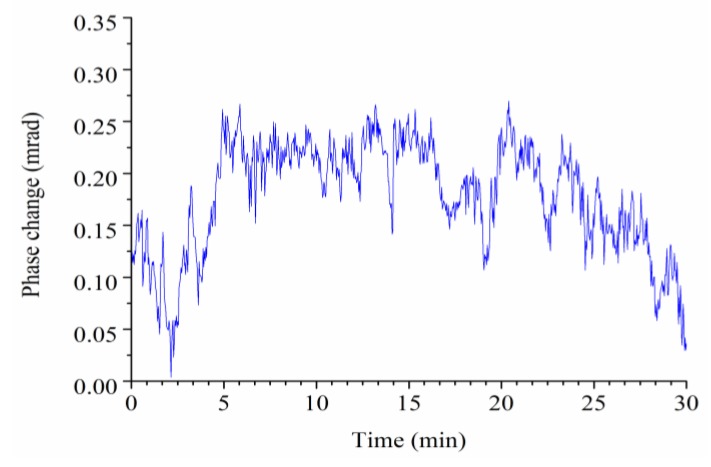
The phase dynamics detected only with the injection of PBS buffer within a 30-min duration.

For evaluating the stability of the phase characterization, we first measured the phase dynamics only with the injection of PBS buffer (without interactive or binding molecules) within 30 min. As shown in Figure 5, the total dynamic range of the measured phase, within the 30-min duration, was less than 0.27 mrad (thickness of 28 pm in air). This demonstrated that the biosensor has excellent phase stability. 

**Figure 6:**
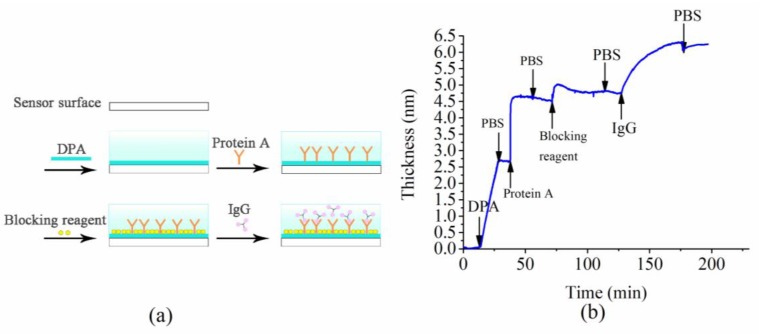
(**a**) A schematic diagram of describing the procedures of probe functionalization and molecular binding. (**b**) The thickness dynamics induced by the functionalization of probe and binding of target IgG. The concentration of IgG solution is 30 µg/mL. DPA—dopamine. PBS—phosphate buffered saline solution. IgG—immunoglobulin G.

To demonstrate the capability of the developed fluidic biosensor in real-time molecular detection, the biosensor was used for detecting IgG molecules (rabbit IgG solution, 30 µg/mL). Figure 6a shows the procedures of molecular modification and binding. IgG molecules are specifically binding on the protein A modified probe surface. The change in thickness of the molecular layer during the procedures of molecular modification and binding is illustrated in Figure 6b. The first three stages exhibiting a dramatic increase in thickness at the 14th, 38th, and 71st minute after data acquisition correspond to the deposition of dopamine, protein A, and blocking reagents, respectively. The last thickness increase, which appeared more progressive, was induced by the specific binding of IgG molecules. The presented biosensor is limited in detecting molecules that can have strong rearrangements of their tertiary structures. Changes in tertiary structures of the target molecules may alter the dimensions of the target molecules and, thus, affect the measurement results [24,25]. However, the changes in molecular dimension due to the alterations in the tertiary structure are insignificant when compared with the dimensions of the target molecules. As a result, our system and method are still valid when detecting molecules with specific tertiary structures.

**Figure 7:**
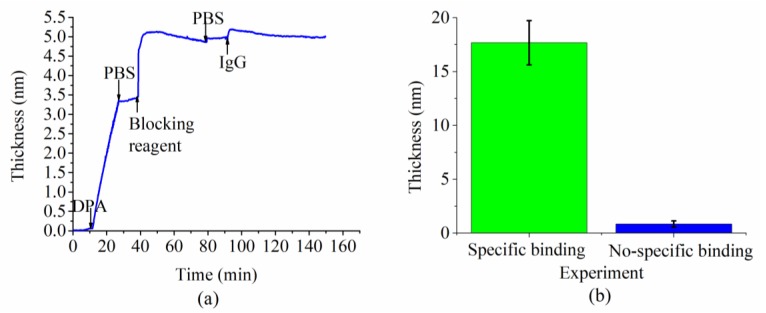
(**a**) The thickness dynamics induced by the probe functionalization and binding of target IgG in a nonspecific binding experiment. The concentration of IgG solution is 30 µg/mL. (**b**) The contrast in the thickness changes between the specific binding and non-specific binding experiments.

To further verify the last progressive increase in the thickness of the molecules layer was only generated by the specific binding of IgG molecules to the probe molecules protein A (without nonspecific absorption on the probe surface), we performed another nonspecific binding experiment, which differed from the specific binding experiment by removing the protein A. The protein-free blocking reagent were injected into the flow channel directly after the deposition of the dopamine layer. The detected thickness dynamics revealed a larger thickness increase induced by the injection of blocking reagent molecules (Figure 7a), which could be explained by the fact that more vacant binding sites were exposed on the dopamine layer without the occupation from protein A molecules and, therefore, more blocking reagent molecules were binding onto the dopamine layer. The last thickness increase corresponding to the specific binding of IgG molecules in the specific binding experiment was replaced by a much smaller and steeper thickness increase in this nonspecific binding experiment. This smaller thickness change indicated that the IgG molecules were rarely captured in this solution due to the absence of their probe molecules known as protein A. The contrast in this thickness increase between these two experiments (Figure 7b) proved that the progressive thickness increase appearing in the specific binding experiment with the presence of protein A was generated by the specific binding of antibodies IgG molecules to the probe molecules protein A. Hence, the capability of the developed fluidic biosensor in analyzing molecular activities such as specific molecular recognition was further demonstrated.

**Figure 8:**
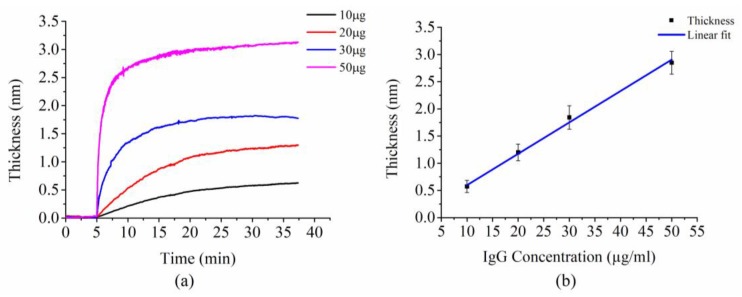
(**a**) The thickness dynamics corresponding to binding of target IgG at concentrations of 10, 20, 30, and 50 µg/mL. (**b**) The linear relationship between the thickness change and the concentration of the target IgG solution. Each plotted data point was averaged from three measurements. The blue solid line is the linear fit.

In addition to the determination of specific molecular recognition, the sensitivity of this developed biosensor was estimated. The thickness increase induced by the specific binding of IgG molecules to protein A molecules appeared to be linearly proportional to the concentration of IgG (Figure 8). The coefficient of variation for the concentration of 10, 20, 30, and 50 μg/mL are 0.196, 0.128, 0.116, and 0.073, respectively. The slope of the linear fit was calculated to be 0.056 nm/(µg/mL). The minimum detectable molecular concentration, which indicates the sensitivity of this developed biosensor, can be calculated as $3\sigma_{s}/\left(\delta h/\delta c\right)$ where $\sigma_{s}$ is the standard deviation of the thickness after binding completion and δh/δc is the slope of the thickness-concentration curve. The standard deviation of the thickness from the data of 10 µg/mL is calculated as 0.006 nm. Consequently, the sensitivity of this developed biosensor was calculated to be 0.11 µg/mL.

## 4. Conclusions

In conclusion, the fluidic biosensor based on a phase-sensitive SD-LCI has been demonstrated to be capable of real-time and label-free monitoring of molecular activities such as binding reactions. Such monitoring is conducted through phase analysis of the specific interference signal from the probe surface, which enables the subsequent detection and measurement of the subnanometric changes in the thickness of the molecular layers. Future research would refer to the improvements enabling multi-point detection, which may require multiple reaction cavities and fiber optic probes with coverslips of different thicknesses and, thus, simultaneously provide both pre-reaction and post-reaction information and further miniaturization of the fiber optic probe, which may require replacement of the ceramic ferrule with a thinner glass capillary.
